# Trade-off between growth, nutrient absorption and medicinal quality of *Sarcandra glabra* (Thunb.) Nakai under different Chinese fir-based agroforestry systems

**DOI:** 10.3389/fpls.2026.1868306

**Published:** 2026-07-01

**Authors:** Daocheng Ma, Fengjing Jiang, Jiayan Wang, Meng Liang, Mingye Huang, Weibin Meng, Yuanyuan Xu, Mei Yang

**Affiliations:** 1 Guangxi Colleges and Universities Key Laboratory for Cultivation and Utilization of Subtropical Forest Plantation, Guangxi University, Nanning, China; 2 School of Forestry, Guangxi University, Nanning, China; 3 Guangxi State-Owned Gaofeng Forest Farm, Nanning, Guangxi, China

**Keywords:** agroforestry systems, chemical components, comprehensive analysis, growth and nutrient accumulation, *Sarcandra glabra* (Thunb.) Nakai, soil properties

## Abstract

**Introduction:**

*Sarcandra glabra* (Thunb.) Nakai-*Cunninghamia lanceolata* (Lamb.) Hook. based agroforestry systems achieved high understory space utilization rate and economic values in southern China and more subtropical areas.

**Methods:**

To better understand the soil physio-chemical properties and nutrients/chemical components of *S. glabra* under pure and mixed *C. lanceolata* forests, the growth status, soil properties and some specific components (flavonoids and phenolic acids) of *S. glabra* were determined under pure *C. lanceolata* forest (CS system), *C. lanceolata-Illicium verum* Hook. f. mixed forest (CIS system) and *C. lanceolata-Quercus griffithii* Hook. f. & Thomson ex Miq. mixed forest (CQS system).

**Results:**

*S. glabra* under CQS system had the best growth status, while it showed lower growth and nutrient status under CIS system. Nutrient accumulation of leaves was dominant among organs, C and K accumulated more in CIS and CQS than CS system, while N and P were opposite. As for medicinal properties, *S. glabra* in CIS system had the highest total flavonoid and chlorogenic acid contents, while isofraxidin and rosmarinic acid contents reached the maxima in CQS system. Non-rhizosphere soil under CIS system had higher moisture-holding capacity and less porosity than the other two systems, CQS and CIS systems had higher medicinal value of *S. glabra* seedlings and C, P content in rhizosphere soil than in non-rhizosphere soil. There were negative correlations between chemical components and most of the plant nutrient contents, while positive correlations existed between different chemical component contents, speculating that CQS systems could be recommended for maximizing yield and some specific phenolic acids, while CIS systems was suitable for flavonoid accumulation.

**Discussion:**

The yield, nutrient accumulation and medicinal quality of *S. glabra* under mixed *C. lanceolata* forest should be balanced by proper intercropping and cultivation methods.

## 1 Introduction

“Bionic wild” agroforestry systems for medicinal plants has been widely practiced since the early 2000 s in southern and southwestern China, making efficient use of understory spaces and the protection of forest ecosystems sustainability (Li et al., 2025a; Chen et al., 2022). Appropriate understory cultivation of medicinal plants could enhance the growth status of both upper forest and understory crops and also play an important role in the improvements of soil texture and water conservation (Zhu et al., 2018; Qiao et al., 2020). For example, the understory cultivation of *Sophora davidii* Kom. ex Pavol. and *Thermopsis lanceolata* R. Br. could enhance the growth status and economic incomes of *Xanthoceras sorbifolium* Bunge via strong N-fixing abilities and the improvements of soil-plant environments (Wang et al., 2019). In addition, soil comprehensive physio-chemical status of upper forests could be enhanced after proper intercropping, with higher soil moisture content and carbon, nitrogen and phosphorus fertility. Upper-layer forest could also promote understory medicinal plants to some extents, like *Bletilla striata* (Thunb. ex A. Murray) Rchb. f. -*Carya cathayensis* Sarg (Cao et al., 2024), *Pinus massoniana* Lamb.*-Curcuma longa* L (Ullah et al., 2024). and many other agroforestry systems. The medicinal quality of understory crops could also be enhanced by nutrient competition, redistribution and the changes of ecological indicators after understory cultivation (Isaac and Borden, 2019; Ma et al., 2026), such as higher saponin and nutrients contents in *Panax notoginseng* (Burkill) F. H. Chen ex C. H. Chow-*Platycladus orientalis* (L.) Franco/*Schima wallichii* (DC.) Korth. systems (Wang et al., 2025), higher polysaccharides and total flavonoid contents of *Polygonatum odoratum* (Mill.) Druce -*Vernicia fordii* (Hemsl.) Airy Shaw system (Zhou et al., 2025) and others. In summary, suitable “bionic wild” agroforestry systems were beneficial to both upper forest and understory medicinal plants, which could also be greatly improved in actual productions.

Chinese fir (*Cunninghamia lanceolata*) is one of the major timber trees in southern China (for more than 1000 years of artificial cultivation) and various (sub) tropical regions, with 24% artificial forest plantation area in China and 6.1% in the world. Due to land degradation and growth limitations, it is urgent to improve the low-production pure *C. lanceolata* forest by mixed planting, intercropping or proper nurturing methods (Li et al., 2023a). Understory cultivation was commonly used because of its high renovation efficiency, short harvesting cycles and environmentally-friendly methods. The mixed-planting of *Triticum aestivum* L., *Zea mays* L. and other economic crops could prevent gradual depletion and degradation of soil nutrients, even improve the growth status by improving microbial communities and available nutrients releasing (Wang et al., 2005). The key enzyme activities and soil fertility could be better improved during mixed-plantation process of leguminous species (such as *Dalbergia balansae* Prain and *Spatholobus suberectus* Dunn) (Zhang et al., 2020; Guo et al., 2023). Moreover, the yield and contents of epiberberine, palmatine, and berberine of *Coptis chinensis* Franch. could be enhanced under cultivated in *C. lanceolata* forests (Duan et al., 2024). In recent years, *Illicium verum* and *Quercus griffithii* were used to mixed-planting with *C. lanceolata* because of their wide cultivation area, good economic values and strong adaptation to the environment in Guangxi (Ming et al., 2019; Chen et al., 2024). However, the differences of their allelopathic and understory potentials to the understory medicinal plants should be further studied in order to build the basics for the foundation of “*C. lanceolata*-based” agroforestry systems. Exploring a suitable agroforestry system is important to make full use of the understory space of Chinese fir forests and improve its forest ecosystems.

*Sarcandra glabra* (Family Chloranthaceae, named “Caoshanhu” in Chinese) is an evergreen hemi-shrub that mainly inhabits in Guangxi Zhuang Autonomous Region (southern China) and many other Asian and African countries. Abundant flavonoids (Qin et al., 2024), polyphenols (Petersen and B€omeke, 2025) and other chemical components in its leaves and whole plants showed strong medicinal and antioxidant values. Due to the high demand and shade tolerance, *S. glabra* have been cultivated artificially in various (sub) tropical understory regions (Zeng et al., 2021). Previous research showed that proper provenances introduction of *S. glabra* from Guangxi and Jiangxi could better adapt to the cultivation environment in Hunan province, which was mainly related to similar environment between its original habitats and cultivation environment (Nie et al., 2021). It is necessary to explore reasonable understory cultivation methods to enhance its yield and medicinal quality. Except for cultivation environment, proper allelopathic effects, interspecies relationships and cultivation methods could also enhance the yield and quality of *S. glabra.* For example, the addition of *Liriodendron × sinoamericanum* P. C. Yieh ex C. B. Shang & Zhang R. Wang leaf powder showed positive allelopathic effects on the growth, physiological status, microbiological structure, and specific chemical component contents of *S. glabra* (Wu et al., 2022). “*C. lanceolata- S. glabra*” agroforestry system could improve the soil carbon content in the upper layer and the economic income of the agroforestry system than *C. lanceolata* and *Phyllostachys edulis* (Carrière) J. Houz. pure forest (Zhou et al., 2022; Xu et al., 2024). The intercropping of *S. glabra* under *C. lanceolata* forest could promote soil properties by changing the volatile organic compounds (VOCs, including phenols, organic acids and hydrocarbon compounds, which were low-polarity, low-boiling and easily spreadable) in its root exudates and soil enzyme activities in forest soil (Jia et al., 2025). In summary, soil fertility and environment of upper forest could be better improved after *S. glabra* intercropping. However, *C. lanceolata* was commonly mixed-planted by many local tree species (like *Michelia macclurei* Dandy, eucalyptus, etc.) in recent years, with better conditions of soil characteristics (Deng et al., 2024). Previous studies mainly focused on the relationships between *C. lanceolata* and *S. glabra* pure stands and single traits (growth or soil carbon), and the understanding of the interactions between mixed *C. lanceolata* forests (like *I. verum*, *Q. griffithii* and others mentioned in the previous section) and *S. glabra* agroforestry systems of plant growth, nutrients absorption and medicinal components accumulation was lacking. Therefore, selecting suitable upper *C. lanceolata* forest-whether pure or mixed with different companion species-is therefore critical for optimizing *S. glabra* understory cultivation and maximizing economic returns in Guangxi.

This study explored the comprehensive effects of growth, nutrient contents, soil properties and medicinal components of 24-month-old *S. glabra* seedlings under pure *C. lanceolata* forest (CS system) and *C. lanceolata-I. verum/Q. griffithii* mixed forest (CIS and CQS system), and aimed to address the following scientific questions: (1) How did different upper *C. lanceolata* forests influence the growth, yield and medicinal quality of *S. glabra* seedlings? (2) What were the differences of comprehensive status between different agroforestry systems? (3) What is the relationship between growth, yield, and the status of nutrients and chemical components in *S. glabra* seedlings? We hypothesized that: (1) *S. glabra* showed better conditions under mixed *C. lanceolata* forest that pure forest; (2) Leaf was the main nutrient accumulation organ for *S. glabra*, and mixed forest might enhance the nutrients absorption; (3) There was a trade-off between yield and medicinal quality, with growth and nutrient accumulation being strongly correlated. Our findings provide a scientific basis for selecting optimal understory configurations for *S. glabra* cultivation in *C. lanceolata* plantations.

## 2 Materials and methods

### 2.1 Test site

The experiment was conducted in Guangxi State-Owned Gaofeng Forest Farm, Nanning City, Guangxi Zhuang Autonomous Region, China (108°08′–108°53′ E, 22°49′–23°15′ N). The average annual temperature, precipitation, and annual relative humidity were 21.6°C, 1643.00 mm and 79.00%, respectively, with subtropical monsoon climate, which is suitable for the growth of *S. glabra* and test forests (Lu et al., 2025).

### 2.2 Plant materials

The 24-month-old seedlings of *S. glabra* were provided by Guijie Agricultural Technology Co., LTD (in Long’an County, Guangxi Zhuang Autonomous Region, P.R. China). *S. glabra* was a kind of common understory medicinal plant which inhabited in Guangxi and Southern China (which was out of the IUCN Red List of Threatened Species). The seeds of *S. glabra* were obtained from Guangxi Medicinal Botanical Garden with permission from the garden. No endangered or protected wild populations were sampled. The material transference and cultivation complied with local regulations and applicable ethical guidelines for plant genetic resources. After germination, the seedlings were transplanted into small plastic pots (15 cm in radius and 20 cm in height) with proper nutrient and water management (plant height was approximately 20 cm).

### 2.3 Experimental design

All three types of forest were planted in 1996, with an average altitude and slope were 485 m and 20°, respectively. The soil layer was thicker than 80 cm, and the initial planting density was 2500 plants per hectare (with planting space of 2 m×2 m), with three thinning in 2003, 2008, and 2013. A completely randomized block experiment was conducted in April 2019. Twenty-four-month-old *S. glabra* seedlings were transplanted with soil balls under three types of forests: *C. lanceolata* pure forest, *C. lanceolata-I. verum* mixed forest and *C. lanceolata*-*Q. griffithii* mixed forest (which were treated as CS, CIS and CQS respectively, with three 20 m×20 m sample plots as biological replicates (Totaling 9 sample plots. The plots were spatially independent and there was a distance of about 50 m between different sample plots). The understory canopy densities of the three kinds of forests were ranging from 0.75 to 0.80 (with no significant differences). The planting density of *S. glabra* in them was all 10000 plants per hectare (with planting space of 1 m×1 m). When planting *S. glabra* seedlings, 750 kg of compound organic and inorganic fertilizer (provided by Guangxi Gaolin Fertilizer Industry Co. Ltd, N:P_2_O_5_:K_2_O=15:6:9, with 10% organic matters) was added per hectare as the based fertilizer (75 g per plant, according to Yang (2012) and the experience of understory *S. glabra* cultivation gained from production practices accordingly). Weeding was performed six times each year (without any other additional measures). *S. glabra* seedlings were harvested in December 2022 (after 3.5-year cultivation). The status of growth, biomass accumulation, soil and plant nutrition, and chemical component contents were determined.

### 2.4 Indices determination

#### 2.4.1 Aboveground growth

In each biological replicates sample plots, twenty *S. glabra* seedlings were chosen randomly for aboveground growth determination. Plant height was determined by steel tape rulers (as the straight distance from the junction between plants and the ground to the terminal buds, with an accuracy of 0.01 cm); Ground diameter was determined by electronic vernier calipers (as the thickness at the junction between plants and the ground, with an accuracy of 0.01 mm).

#### 2.4.2 Biomass accumulation

Three healthy *S. glabra* seedlings were randomly selected for each sample plots (and the average of them was treated as a biological replicate, and a total of three biological replicates were set in an agroforestry system). All the seedlings were divided into separate organs (roots, stems and leaves), cleaned tap water and deionized water were used to wash them in order. Fresh weights were determined immediately after wiping clean (within 0.01 g). After fresh weight determination, all the organs were sterilized at 105°C for 30 min and then dried at 75°C to constant weight (within 0.01 g) (Ma et al., 2024).

#### 2.4.3 Plant nutrient contents and stoichiometric ratio determination

The dried roots, stems, and leaves samples were grinded and passed through the 60-mesh sieve. The H_2_SO_4_-H_2_O_2_ boiling method was used for sample treatments. An AA3 continuous flow analyzer, molybdenum-antimony resistance colorimetric method, and flame photometer were used to determine total nitrogen (TN), total phosphorus (TP), and total potassium (TK) contents, respectively. Besides, the stoichiometric ratios of TC/TN, TC/TP, TN/TP, TN/TK and TK/TP were calculated for nutrient limitation analysis (Ma et al., 2026).

#### 2.4.4 Plant chemical component contents

Dried samples in 2.4.3 section were also used to determine total flavonoid and some specific phenolic acids contents. Detailed information was listed as follows:

(1) Total flavonoid determination: The determination of total flavonoid content was referred to Lima et al. (2024), which was listed as follows:

1. Samples extraction: About 0.2 g dried samples were put in 50 mL plastic falcon tubes with 20 mL 75% (v/v) ethanol. The mixture was then extracted in a hot bath at 60 °C for 30 min. After extraction, the liquid supernatant from each sample was used for the determination of chemical components.
2. Contents determination: 25 μL extracts were added into plastic falcon tubes and mixed with 100 μL distilled water and 7.5 μL of 5% NaOH (aq). 7.5 μL 10% AlCl_3_ (aq) was added to the mixtures after 6 min reaction and then incubated for 6 min. 100 μL 4% NaOH (aq) and 10 μL deionized water were added and reacted for 15 min. The mixed liquid supernatants were used for color comparison at 510 nm wavelength using an ultraviolet spectrophotometer (UV-2450; Shimadzu, Tokyo, Japan) after a 15 min reaction, which was determined from a standard curve (0–200 μg/mL).

(2) Specific phenolic acids determination: The contents of chlorogenic acid, isofraxidin and rosmarinic acid were determined by UHPLC (Ultra-High Performance Liquid Chromatography)-Q-Exactive Orbitrap MS (Mass Spectrometry) (Thermo Fisher Scientific, USA) according to the methods of Pan et al. (2017) and Nie et al. (2021) (with some improvements):

1. Chemical components extraction: About 1.0 g dried samples were put into 50 mL plastic falcon tubes with 25 mL 50% (v/v) methanol. The mixture was then extracted in a hot bath at 50 °C for 10 min. After cooling to room temperature, 50% (v/v) methanol was used to make up the missing weight. Then the supernatant was passed through a 0.22 μm microporous filter membrane, which was used for chemical components determination.
2. Standard solution preparations: The standard substances of chlorogenic acid, isofraxidin, and rosmarinic acid were precisely weighed 0.01 g and dissolve with methanol, separately. The mixtures were made up the volume to 10 mL and 1 mL if it was taken and made up the volume to 10 mL (the concentration of them were all 100 μg/mL). The standard curve of chlorogenic acid and isofraxidin was 0–40 μg/mL, while the standard curve of rosmarinic acid was 0–100 μg/mL.
3. UHPLC conditions: Chromatographic separation was performed on an Agilent C18 column (250 mm × 4.6 mm, 5 μm) at 35 °C. Acetonitrile (A) and 0.2% phosphoric acid aqueous solution (B) were the mobile phase (with a flow rate of 1.0 mL·min^-1^). The samples injection volume was 10 μL and the gradient elution programs were listed as follows: 0–10 min, 15% A; 10–18 min, 15% → 25% A; 18–30 min, 25% A. The detection wavelengths were set at 344 nm for targeted compound detection.
4. Precision, reproducibility and stability test: The mixed reference substance solutions were taken to inject three times according to the UHPLC conditions. The peak areas were recorded and RSDs (Relative Standard Deviations) of chlorogenic acid, isofraxidin, and rosmarinic acid were calculated (n=3). Then 1.0 g (precisely weigh) of the same dried *S. glabra* powder (in three portions) were weighed to preparing for the test solutions according to chemical components extraction method. All of the extracts were taken to inject three times according to the UHPLC conditions (then the peak areas were recorded). Calculate the RSD of the three components. At last, the same test solution was injected it at 0, 8, 12, and 24 hours respectively according to the UHPLC conditions (then the peak areas were recorded), indicating that the above components were stable in the test solution within 24 hours. The correlation coefficients (R²) for all target compounds were > 0.99. The method showed good precision with RSD values < 3% for all compounds (n = 3). Repeatability RSDs were < 2% (n = 3). The method showed acceptable precision, repeatability, and stability (RSD < 3%), and the average recoveries met the acceptance criteria for plant secondary metabolite analysis.

#### 2.4.5 Soil physical and chemical properties

For each sample plot, three ring knife (volume V = 100 cm^3^) were used to collect soil samples from the 0─20 cm and 20─40 cm layers. The soil moisture content, bulk density, and other indices were calculated by weight determination according to the Chinese national standard (NY/T 52-1987) and Wang et al. (2021) methods. After physical properties determination, non-rhizosphere soil and rhizosphere soil from different soil layers and systems were collected. Non-rhizosphere soil from five points were randomly selected and collected in each sample plot, and all of them were uniformly mixed to form the biological replicate. Then six *S. glabra* plants were randomly selected in each plot for rhizosphere soil collection (which was carefully brushed off the soil tightly adhering to the root surface using a clean brush). The samples of (non) rhizosphere soil were collected into clean plastic bags (about 50 g per replicate), and there were three biological replicates for each system. All of them were air-dried and passed through a 100-mesh sieve. The Multi N/C3100 TOC system was used for total carbon (TC) determination; H_2_SO_4_-CuSO_4_/K_2_SO_4_ boiling and AA3 type continuous flow analyzer (Bran + Luebbe, Hamburg, Germany) were used for total nitrogen (TN) determination, H_2_SO_4_-HClO_4_ boiling was used for total phosphorus (TP) and total potassium (TK) determination, with molybdenum-antimony anti-colorimetric method for TP and flame photometry for TK (Ma et al., 2026).

### 2.5 Statistical analysis

The data were all organized by Microsoft Excel 2016. The results of Shapiro–Wilk test and Levene’s test showed that all the data met the assumptions of ANOVA (Analysis of Variance). One-way variance analysis (with Duncan’s multiple comparisons test, *P* = 0.01) and principal component analysis (PCA) were all performed by IBM SPSS 18.0. As for PCA, all the data were standardized before analysis. Principal components (PCs) were extracted based on the criterion of “eigenvalue > 1”, and the component loadings and PC scores were calculated. Besides, the linear-regression analysis between biomass accumulation and chemical component contents (including total flavonoid and phenolic acids content) were also analyzed by IBM SPSS 18.0. The linear-regression results (R², Pearson’s r, and *P*-values) could be used to assess the “trade-off” strategy of understory *S. glabra.* Microsoft Excel 2016 was used to draw the tables and figures throughout the manuscript. In addition, a correlation heat map was also created by Microsoft Excel 2016 (Ma et al., 2026).

## 3 Results

### 3.1 Aboveground growth and plant biomass accumulation

**Figure 1:**
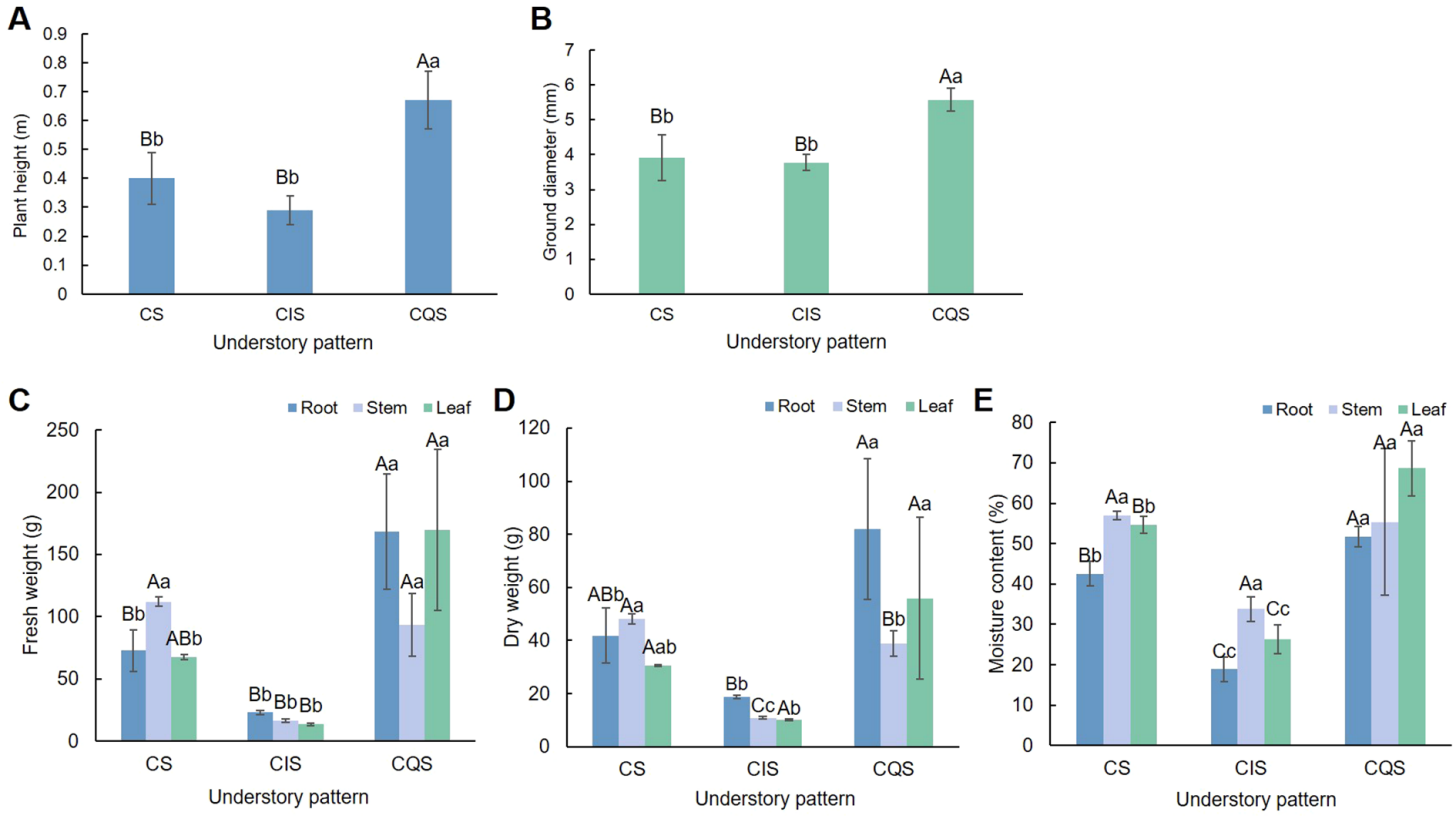
Aboveground growth and biomass accumulation characteristics of *S. glabra* under different agroforestry systems. [**(A)** plant height; **(B)** Ground diameter; **(C)** Fresh weight; **(D)** Dry weight; **(E)** Moisture content]. The length of error bars in the figure represented standard deviation (SD). Different uppercase letters indicated that the values between different agroforestry systems had highly significant differences (*P* < 0.01), while different lowercase letters indicated that the values between different agroforestry systems had significant differences (*P* < 0.05). The same below.

There were highly significant differences in the height and ground diameter of *S. glabra* between the different agroforestry systems (*P* < 0.01, **Figures 1A, B**; **Supplementary Table 1**). Both plant height and ground diameter reached their maxima under CQS system (0.67 m and 5.58 mm, respectively), which were 67.50% and 42.71% higher than those under CS system. In addition, the growth status of *S. glabra* under CIS systems were lower than those in CS and CQS systems, but there were no significant differences between CIS and CS systems. Therefore, the *S. glabra* seedlings had much better growth status in *C. lanceolata-Q. griffithii* mixed forests than those in the other two systems, and *C. lanceolata-I. verum* mixed forest had negative effects on *S. glabra* seedlings. As for biomass accumulation, there were significant differences in the fresh and dry weights of roots, stems, and leaves between different agroforestry systems of *S. glabra* except for leaf dry weight, whereas the moisture content had highly significant differences in roots and leaves between different agroforestry systems (*P* < 0.01, **Figures 1C–E**; **Supplementary Table 2**). As for root and leaf, the fresh weight, dry weight and moisture content all reached the maxima under CQS system (168.57 g, 82.10 g and 51.71% in root, respectively; 169.67 g, 55.93 g and 68.66% in leaf, respectively), while these indices of stem reached the maxima in CS system (112.07 g, 48.23 g and 56.96%, respectively). All the indices in CIS system were the lowest among all the agroforestry systems. For biomass accumulation, all the indices of root and leaf were much higher in CQS system than CS and CIS, but the trend in stems showed the opposite trend (CS was much higher than the others). In summary, *S. glabra* under CQS system showed better comprehensive biomass accumulation performances, whereas it had the highest stem biomass under CS system.

### 3.2 Plant nutrient contents and stoichiometric ratios

**Figure 2:**
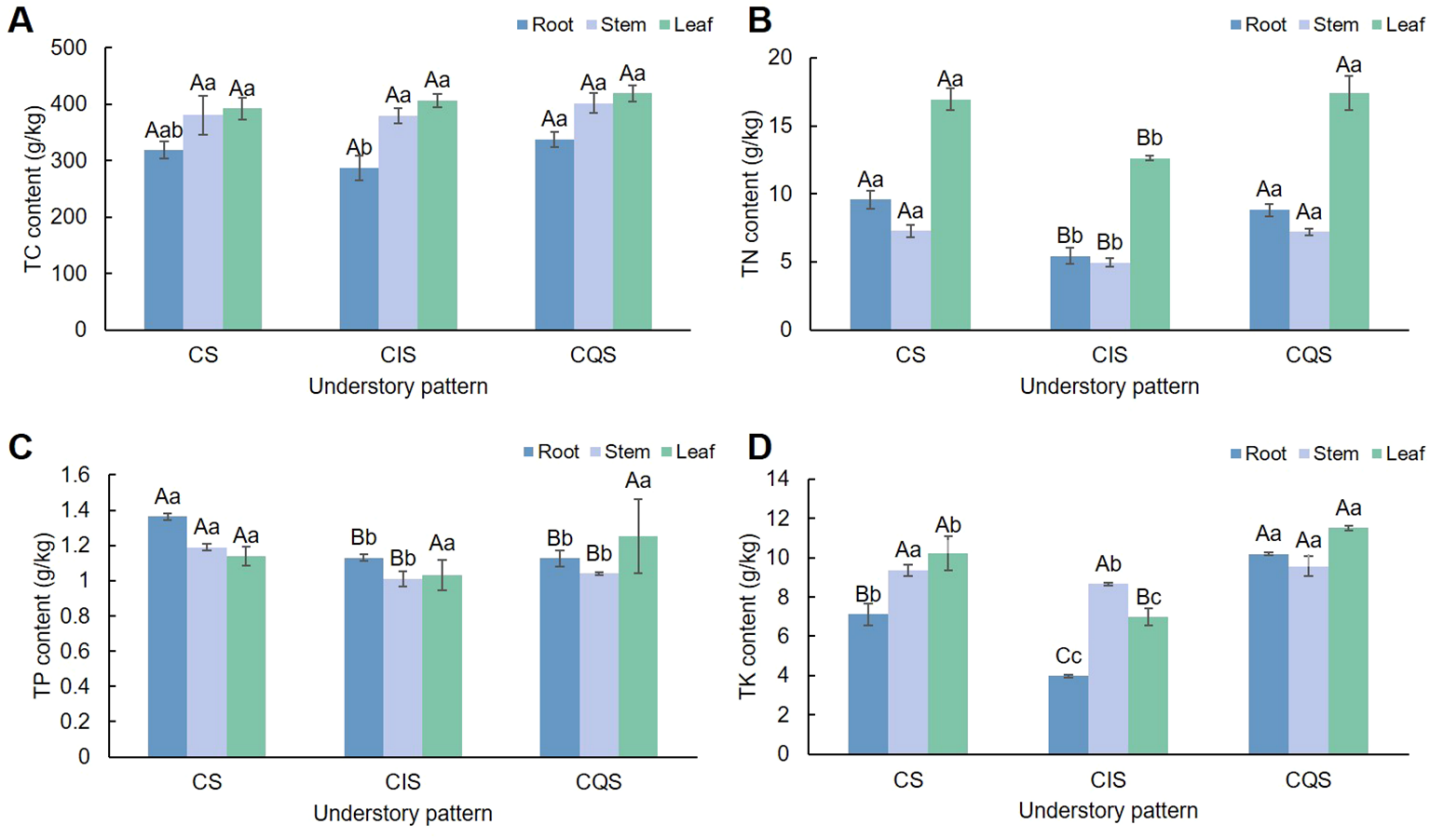
Nutrient contents in different organs of *S. glabra* under different agroforestry systems [**(A)** total carbon (TC) contents; **(B)** total nitrogen (TN) contents; **(C)** total phosphorus (TP) contents; **(D)** total potassium (TK) contents]. Different uppercase letters indicated that the values between different agroforestry systems had highly significant differences (P < 0.01), while different lowercase letters indicated that the values between different agroforestry systems had significant differences (P < 0.05).

There were significant or highly significant differences in TN and TK content in all vegetative organs between different agroforestry systems. For TC content, there were significant differences in roots, while roots and stems had highly significant differences in TP content (**Figures 2A–D**; **Supplementary Table 3**). TC and TN contents showed a trend of leaf>stem>root and leaf>root>stem in most of the systems, speculating that the leaf was the dominant organ for nutrient accumulation. TC content were all the highest of all the organs in CQS system (337.5 g/kg, 402.17 g/kg and 419.27 g/kg), and they were all higher than CS system. TN content were the highest of root and stem in CS system (9.58 g/kg and 7.27 g/kg), while reached the maximum of leaf in CQS system (17.42 g/kg). TP contents reached the maxima of roots and stems in the CS system (1.36 g/kg and 1.19 g/kg), while leaves had the maximum CQS system (1.25 g/kg), while TK contents all reached the maxima in the CQS system (roots: 10.17 g/kg, stems: 9.56 g/kg and leaves: 11.51 g/kg). Therefore, C and K accumulation were improved in the mixed forest system compared to the pure forest system among most the vegetative organs, while N and P showed the opposite trend. The nutrient status in the CQS systems in most of the vegetative organs showed more advantages than the other two systems.

There were significant or highly significant differences in TC/TN and TK/TP in all vegetative organs between different planting systems, and for the other three stoichiometric ratios, there were significant or highly significant differences in roots and stems, except for leaves (**Table 1**; **Supplementary Table 4**). For TC/TN and TK/TP values, the stoichiometric ratios of root, stem, and leaf reached the maxima under the CIS (53.15, 77.22, 32.17) and CQS (9.05, 9.22, 9.37) systems, respectively; For TC/TP, root, stem, and leaf reached the maxima under the CQS, CQS, and CIS systems (300.47, 387.85, 395.79), respectively; For TN/TP, root, stem, and leaf reached the maxima under the CQS, CQS, and CS systems (7.84, 6.95, 14.91), respectively; TN/TK, root, stem, and leaf reached the maxima under the CIS, CS, and CIS systems (1.37, 0.78, 1.81), respectively. TN/TP ratios of roots and stems were lower than 14 under all three systems, and it was also found in leaves under the CIS system, suggesting that these organs had possibly N limitations in these organs and systems. However, this possible interpretation was based on the results of TN measurements reflect total nitrogen contents (including organic forms but not bioavailable nitrate, ammonium and other forms). None of the stoichiometric ratios under the different systems met the conditions of “TN/TP>16” or “TN/TK>2.1 and TK/TP<3.4”, speculating that the limitation effects of P and K were not evident. In summary, N may play an important role in the understory cultivation of *S. glabra* seedlings, but the limitation effects and roles of N requires further addition experiments.

**Table 1:** Nutrient stoichiometric ratio in different organs of *S. glabra* seedlings under different agroforestry systems.

System/organ	TC/TN	TC/TP	TN/TP	TN/TK	TK/TP
CS/Root	33.32 ± 0.84Bb	233.52 ± 13.03Ab	7.02 ± 0.57Aa	1.35 ± 0.02Aa	5.21 ± 0.47Bb
CIS/Root	53.15 ± 5.69Aa	254.28 ± 23.73Ab	4.82 ± 0.61Bb	1.37 ± 0.18Aa	3.52 ± 0.01Cc
CQS/Root	38.32 ± 2.59Bb	300.47 ± 21.18Aa	7.84 ± 0.21Aa	0.87 ± 0.04Bb	9.05 ± 0.33Aa
CS/Stem	52.28 ± 1.49Bb	319.23 ± 25.87Ab	6.10 ± 0.33Bb	0.78 ± 0.03Aa	7.85 ± 0.14Ab
CIS/Stem	77.22 ± 5.41Aa	377.99 ± 22.63Aa	4.90 ± 0.12Cc	0.57 ± 0.04Bb	8.62 ± 0.42Aa
CQS/Stem	55.81 ± 2.67Bb	387.85 ± 17.13Aa	6.95 ± 0.28Aa	0.76 ± 0.07Aa	9.22 ± 0.44Aa
CS/Leaf	23.15 ± 0.76Bb	345.30 ± 28.40Aa	14.91 ± 0.91Aa	1.67 ± 0.23Aa	8.97 ± 0.74Aa
CIS/Leaf	32.17 ± 0.71Aa	395.79 ± 27.28Aa	12.30 ± 0.82Aa	1.81 ± 0.09Aa	6.78 ± 0.16Ab
CQS/Leaf	24.11 ± 0.90Bb	341.68 ± 55.61Aa	14.20 ± 2.53Aa	1.51 ± 0.13Aa	9.37 ± 1.42Aa

The data in the table represent “Mean ± SD (Standard Deviation)”. Different uppercase letters in the same column indicated that the values between different agroforestry systems had highly significant differences (*P* < 0.01), while different lowercase letters in the same column indicate that the values between different agroforestry systems had significant differences (*P* < 0.05).

### 3.3 Flavonoids and some phenolic acids contents

**Figure 3:**
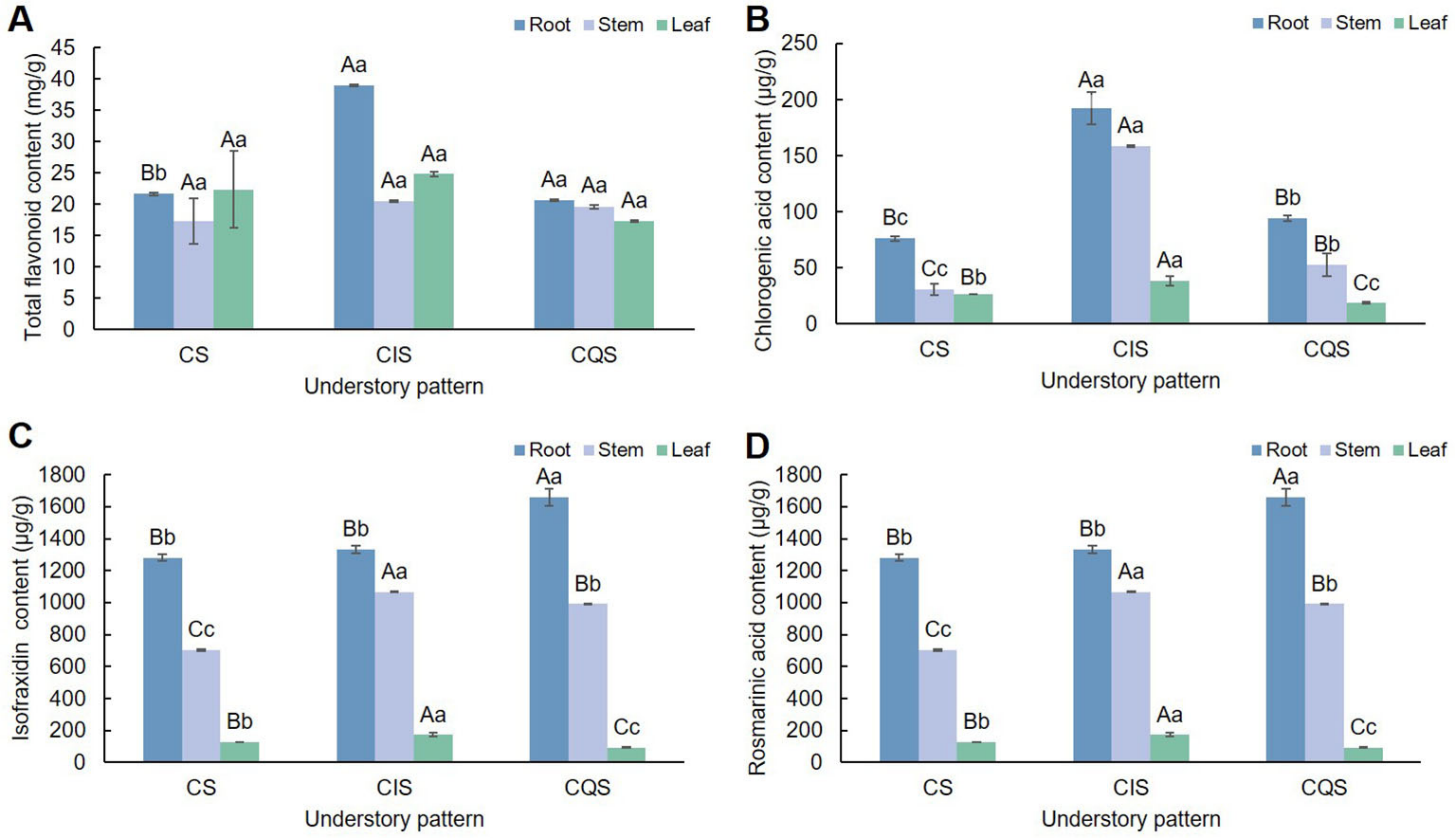
Chemical component contents in different organs of *S. glabra* under different agroforestry systems [**(A)** total flavonoid contents; **(B)** total chlorogenic acid contents; **(C)** total isofraxidin contents; **(D)** total rosmarinic acid contents].

Except for total flavonoid content, there were highly significant differences in chlorogenic acid, isofraxidin, and rosmarinic acid content in roots, stems, and leaves under different planting systems (**Figures 3B–D**; **Supplementary Table 5**), while total flavonoid content only showed highly significant differences in roots (**Figure 3A**; **Supplementary Table 5**). Total flavonoid content reached the maxima under CIS system in root, stem and leaf (38.96 mg/g, 20.48 mg/g and 24.82 mg/g). Chlorogenic acid (192.22 μg/g, 158.51 μg/g and 38.35 μg/g) and rosmarinic acid (122.44 μg/g, 164.81 μg/g and 47.56 μg/g) contents also showed similar trends among root, stem, leaf and all the planting systems. For isofraxidin content, root, stem and leaf reached the maximum under CQS, CIS and CIS systems (1658.21 μg/g, 1067.39 μg/g and 173.08 μg/g), indicating that most of the organs had the highest chemical component contents under CIS systems (root was the main accumulation organ). Mixed forest systems (CIS and CQS) showed better performance than the pure forest agroforestry system (CS).

### 3.4 Soil physio-chemical components

#### 3.4.1 Soil physical properties

Except for maximum water-holding capacity, noncapillary porosity, and total porosity, there were significant or highly significant differences between different planting systems in the 0─20 cm soil layer, whereas in the 20–40 cm soil layer, most of the indices had (highly) significant differences between different planting systems except for volumetric moisture content, soil water storage capacity, non-capillary porosity, and soil permeability (**Table 2**; **Supplementary Table 6**). For the 0–20 cm soil layer, the CIS system had the highest moisture conversion coefficient, soil bulk density, non-capillary porosity, and soil permeability (0.82, 1.39, 0.07, and 0.17, respectively), while the CQS system had the highest mass moisture content, volumetric moisture content, soil water storage capacity, field capacity, capillary porosity, and total porosity (31.96, 40.57, 0.81, 0.32, 0.48, and 0.53, respectively); the CS system had the highest maximum water-holding capacity and capillary moisture capacity (0.44 and 0.39, respectively). For 20–40 cm soil layer, moisture conversion coefficient and soil bulk density had the maxima under CIS system (0.80 and 1.45, respectively); Noncapillary porosity reached the maximum under CQS system (0.07), and the rest indices (mass moisture content, volumetric moisture content, soil water storage capacity, maximum water-holding capacity, capillary moisture capacity, field capacity, capillary porosity, total porosity and soil permeability) were all the highest under CS system (30.45, 38.52, 0.77, 0.43, 0.39, 0.30, 0.49, 0.55 and 0.16, respectively).

**Table 2:** Soil physical characteristics in different layers of soil under different *S. glabra* agroforestry systems.

Agroforestry systems	Moisture conversion coefficient		Soil bulk density		Mass moisture content	
	0–20 cm	20–40 cm	0–20 cm	20–40 cm	0–20 cm	20–40 cm
CS	0.77 ± 0.01Bb	0.77 ± 0.02Ab	1.20 ± 0.07Ab	1.27 ± 0.06Bb	30.22 ± 2.37Aa	30.45 ± 3.54Aa
CIS	0.82 ± 0.02Aa	0.80 ± 0.01Aa	1.39 ± 0.08Aa	1.45 ± 0.03Aa	21.94 ± 2.50Bb	24.28 ± 1.42Ab
CQS	0.76 ± 0.01Bb	0.77 ± 0.01Ab	1.27 ± 0.03Aab	1.29 ± 0.02Bb	31.96 ± 2.11Aa	29.63 ± 1.35Aa
Agroforestry systems	Volumetric moisture content		Soil water storage capacity		Maximum water-holding capacity	
	0–20 cm	20–40 cm	0–20 cm	20–40 cm	0–20 cm	20–40 cm
CS	36.01 ± 0.80Bb	38.52 ± 3.19Aa	0.72 ± 0.02Bb	0.77 ± 0.06Aa	0.44 ± 0.05Aa	0.43 ± 0.01Aa
CIS	30.42 ± 1.82Cc	35.24 ± 1.36Aa	0.61 ± 0.04Cc	0.70 ± 0.03Aa	0.34 ± 0.03Aa	0.34 ± 0.01Bb
CQS	40.57 ± 1.69Aa	38.23 ± 1.24Aa	0.81 ± 0.03Aa	0.76 ± 0.02Aa	0.42 ± 0.03Aa	0.42 ± 0.01Aa
Agroforestry systems	Capillary moisture capacity		Field capacity		Capillary porosity	
	0–20 cm	20–40 cm	0–20 cm	20–40 cm	0–20 cm	20–40 cm
CS	0.39 ± 0.03Aa	0.39 ± 0.02Aa	0.30 ± 0.02Aa	0.30 ± 0.04Aa	0.46 ± 0.01Aa	0.49 ± 0.02Aa
CIS	0.29 ± 0.02Ab	0.31 ± 0.01Bb	0.22 ± 0.02Bb	0.24 ± 0.01Ab	0.41 ± 0.01Bb	0.45 ± 0.01Ab
CQS	0.38 ± 0.03Aa	0.36 ± 0.02Aa	0.32 ± 0.02Aa	0.30 ± 0.01Aa	0.48 ± 0.02Aa	0.46 ± 0.01Aab
Agroforestry systems	Noncapillary porosity		Total porosity		Soil permeability	
	0–20 cm	20–40 cm	0–20 cm	20–40 cm	0–20 cm	20–40 cm
CS	0.06 ± 0.01Aa	0.06 ± 0.02Aa	0.52 ± 0.03Aa	0.55 ± 0.01Aa	0.16 ± 0.02Aa	0.16 ± 0.04Aa
CIS	0.07 ± 0.01Aa	0.05 ± 0.01Aa	0.48 ± 0.02Aa	0.49 ± 0.01Bb	0.17 ± 0.01Aa	0.14 ± 0.01Aa
CQS	0.05 ± 0.01Aa	0.07 ± 0.01Aa	0.53 ± 0.03Aa	0.54 ± 0.01Aa	0.12 ± 0.01Ab	0.15 ± 0.01Aa

The data in the table represent “Mean ± SD (Standard Deviation)”. Different uppercase letters in the same column indicated that the values between different agroforestry systems had highly significant differences (*P* < 0.01) in the same soil layer, while different lowercase letters in the same column indicate that the values between different agroforestry systems had significant differences (*P* < 0.05) in the same soil layer.

#### 3.4.2 Soil pH and chemical properties

**Figure 4:**
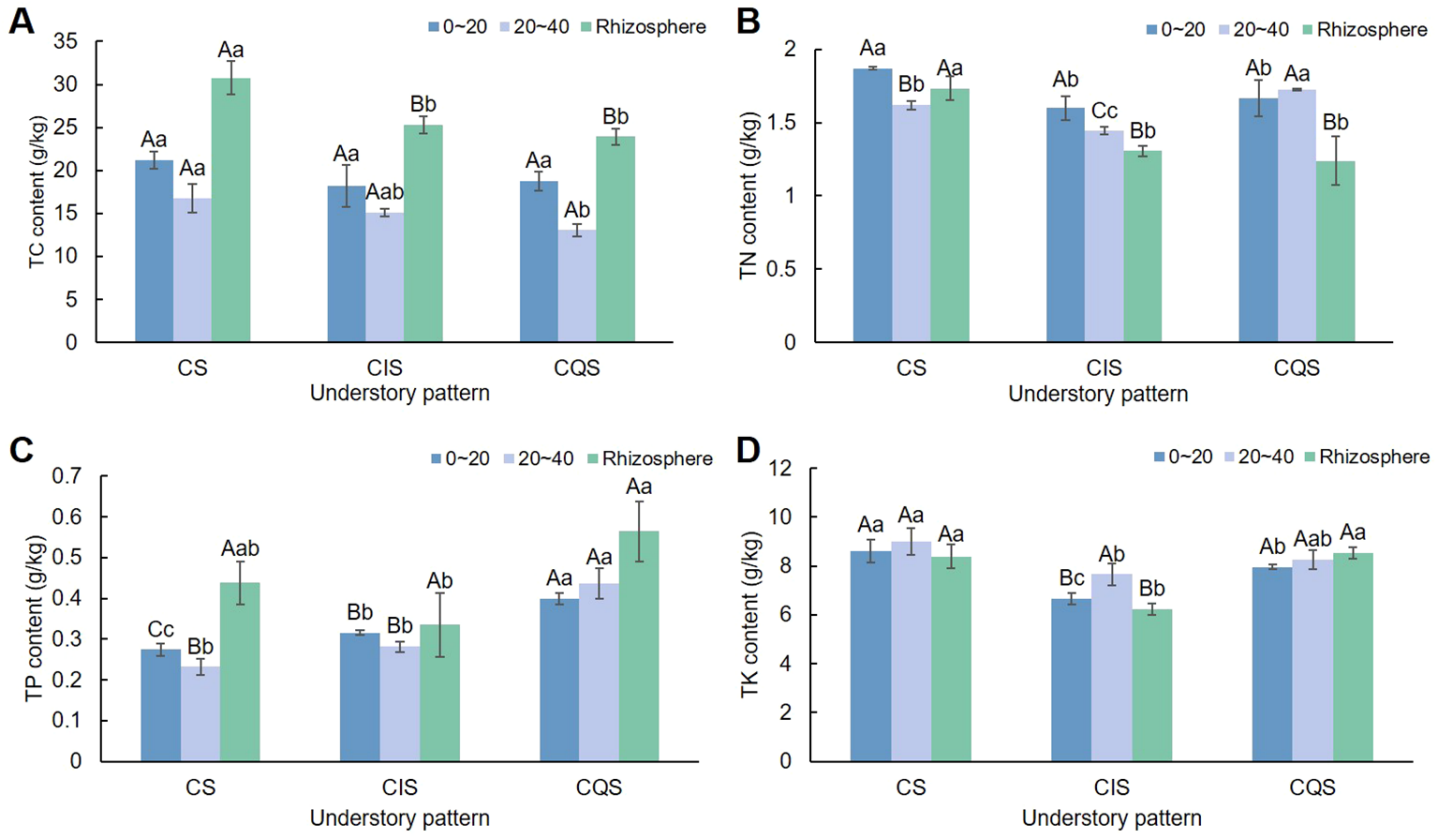
Nutrient contents in different layers of soil under different *S. glabra* agroforestry systems [**(A)** total carbon (TC) contents in rhizosphere and non-rhizosphere soil; **(B)** total nitrogen (TN) contents in rhizosphere and non-rhizosphere soil; **(C)** total phosphorus (TP) contents in rhizosphere and non-rhizosphere soil; **(D)** total potassium (TK) contents in rhizosphere and non-rhizosphere soil].

Except for TC content, there were significant or highly significant differences between different systems in the 0–20 cm soil layer, and there were all significant or highly significant differences between 0–20 cm (20–40 cm) non-rhizosphere soil and rhizosphere soil (**Figures 4A–D**; **Supplementary Table 7**). TC contents of 0–20 cm layer soil, 20–40 cm layer soil and rhizosphere soil were all the highest under CS system (21.17 g/kg, 16.75 g/kg and 30.74 g/kg). For mixed forest systems, TC content of CIS in 0–20 cm soil layer was lower than that in CQS, but 20–40 cm soil layer and rhizosphere soil showed the opposite trend (**Figure 4A**); TN contents reached the maxima in 0–20 cm soil layer and rhizosphere soil of CS system (1.87 g/kg and 1.73 g/kg), while the content in 20–40 cm soil of CQS system reached the maximum (1.72 g/kg). In mixed forest systems, non-rhizosphere soil of CQS showed better performances, while the rhizosphere soil of CIS system showed the opposite trend (**Figure 4B**); TP contents all reached the maxima under CQS system (0.40 g/kg in 0–20 cm soil layer, 0.44 g/kg in 20–40 cm soil layer and 0.56 g/kg in rhizosphere soil), and the status of non-rhizosphere soil in both CIS and CQS systems were better than CS system (**Figure 4C**); TK contents in both two non-rhizosphere soil layers in CS systems (8.61 g/kg in 0–20 cm and 9.00 g/kg in 20–40 cm) were higher, while CQS system had the highest rhizosphere soil content among all the three systems (8.54 g/kg). TK contents in CIS system were lower than another two systems. In summary, mixed forest systems had higher C and P content in rhizosphere soil than in non-rhizosphere soil, C mainly accumulated in the upper soil layer (0–20 cm), N and P contents performed better in CS and CQS systems, respectively, and K mainly accumulated in the deeper soil layer (20–40 cm soil of CS and CIS systems) or rhizosphere soil (CQS system).

For soil stoichiometric ratios, there were significant or highly significant differences in the 20–40 cm soil layer between different planting systems, and in 0–20 cm soil layer, TC/TP, TN/TP, and TK/TP showed highly significant differences, while TC/TP, TN/TP, and TN/TK showed (highly) significant differences between different systems (**Table 3**; **Supplementary Table 8**). For 0–20 cm soil layer, TC/TP, TN/TP, TK/TP all reached the maxima under CS system (77.36, 6.83 and 31.52), while TC/TN, TN/TK reached the maximum under CIS system (11.37 and 0.24); For 20–40 cm soil layer, TC/TP, TN/TP, TK/TP were all reached the maxima under CS systems (72.94, 7.02 and 39.01), while TC/TN, TN/TK reached the maximum under CIS and CQS systems (10.45 and 0.21); For rhizosphere soil, except for TC/TN and TN/TK, CIS system had the highest nutrient stoichiometric ratios of TC/TP, TN/TP and TK/TP (78.01, 4.06 and 19.43), while TC/TN reached the maximum under CQS system (19.45), TN/TK reached the maximum under both CS and CIS systems (0.21, and they had no difference). In summary, different layers of non-rhizosphere soil (0–20 cm and 20–40 cm) showed similar characteristics, whereas rhizosphere soil showed opposite trends.

**Table 3:** Nutrient stoichiometric ratio in different layers of soil under different *S. glabra* agroforestry systems.

System/soil layer	TC/TN	TC/TP	TN/TP	TN/TK	TK/TP
CS/0–20 cm	11.32 ± 0.58Aa	77.36 ± 6.75Aa	6.83 ± 0.34Aa	0.22 ± 0.01Aa	31.52 ± 3.36Aa
CIS/0–20 cm	11.37 ± 1.05Aa	57.73 ± 7.71Bb	5.07 ± 0.30Bb	0.24 ± 0.02Aa	21.11 ± 0.36Bb
CQS/0–20 cm	11.31 ± 1.14Aa	46.93 ± 2.02Bb	4.17 ± 0.42Bc	0.21 ± 0.02Aa	19.92 ± 0.74Bb
CS/20–40 cm	10.33 ± 0.89Aa	72.94 ± 13.83Aa	7.02 ± 0.75Aa	0.18 ± 0.01Ab	39.01 ± 4.61Aa
CIS/20–40 cm	10.45 ± 0.49Aa	53.54 ± 2.45Abb	5.13 ± 0.33Bb	0.19 ± 0.01Aab	27.19 ± 2.37Bb
CQS/20–40 cm	7.59 ± 0.42Bb	30.15 ± 3.88Bc	3.97 ± 0.32Bc	0.21 ± 0.01Aa	19.03 ± 1.68Bc
CS/Rhizosphere	17.79 ± 1.39Aa	70.77 ± 8.59Aa	3.97 ± 0.30Aa	0.21 ± 0.01Aa	19.26 ± 1.38Aa
CIS/Rhizosphere	19.39 ± 1.16Aa	78.01 ± 16.91Aa	4.06 ± 1.07Aa	0.21 ± 0.01Aa	19.43 ± 5.56Aa
CQS/Rhizosphere	19.45 ± 2.04Aa	42.96 ± 6.74Ab	2.25 ± 0.55Ab	0.15 ± 0.02Bb	15.26 ± 1.58Aa

The data in the table represent “Mean ± SD (Standard Deviation)”. Different uppercase letters in the same column indicated that the values between different agroforestry systems had highly significant differences (*P* < 0.01), while different lowercase letters in the same column indicate that the values between different agroforestry systems had significant differences (*P* < 0.05).

### 3.5 Comprehensive analysis

#### 3.4.1 Correlation analysis

**Figure 5:**
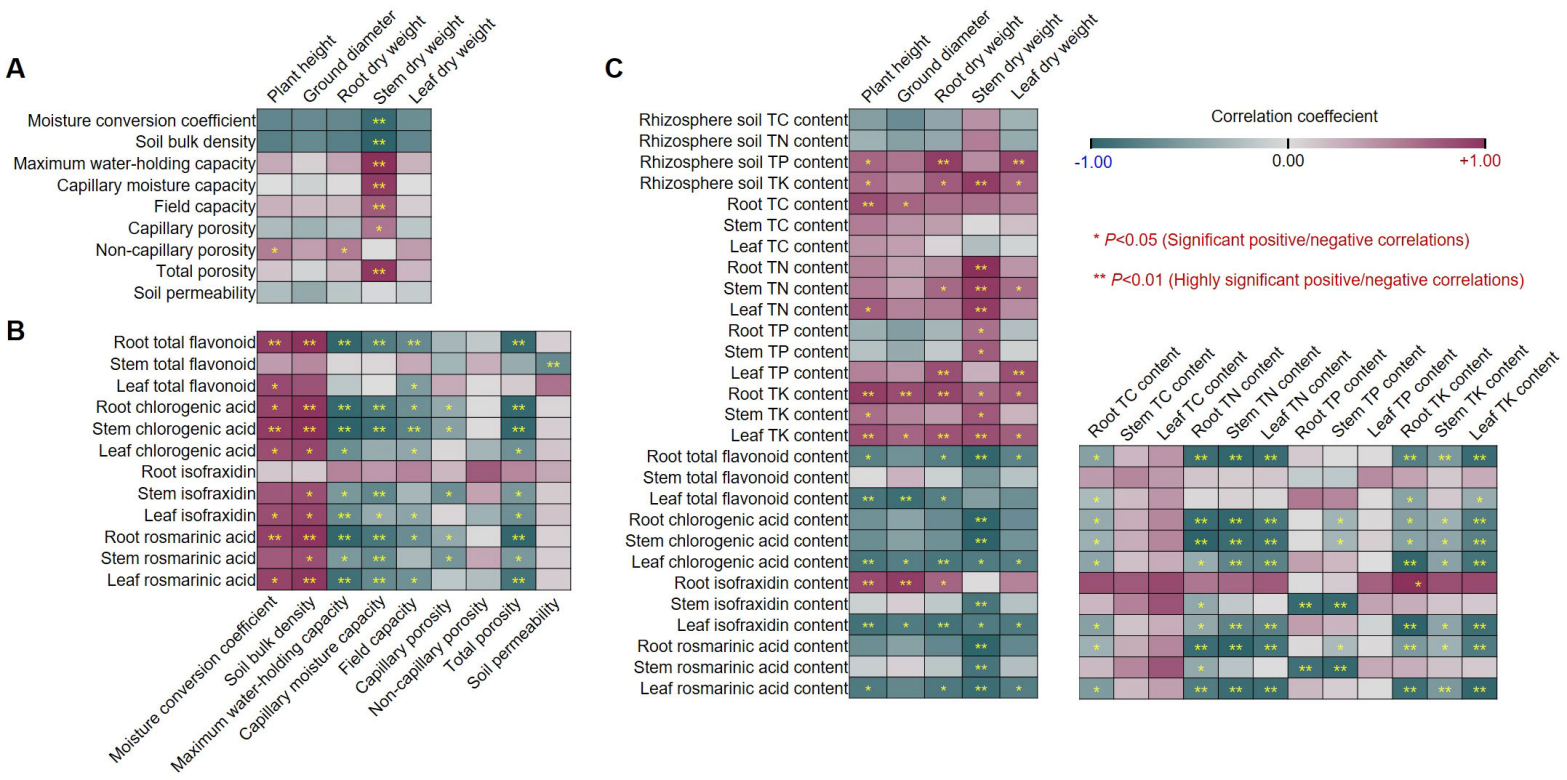
Correlation analysis of *S. glabra* seedlings between different growth, soil physical characteristics, nutrient element accumulations and plant nutrient contents [**(A)** the relationships between the growth, biomass accumulation and soil physical properties; **(B)** the relationships between chemical component contents and soil physical properties; **(C)** the relationships between growth, plant/rhizosphere soil nutrient contents and chemical component contents]. *P<0.05 (Significant positive/neagative correlations), **P<0.01 (Highly significant positive/negative correlations).

There were different correlations between growth, biomass, nutrient and chemical components (**Figures 5A–C**). For seedling growth and soil physical properties, soil non-capillary porosity showed significantly positive correlations with plant height and root dry weight, and stem dry weight showed (highly) significant positive correlations with maximum-water holding capacity, field capacity, total porosity and negative correlations with soil bulk density, suggesting that there were possible associations between soil looseness and the growth condition of *S. glabra* seedlings (especially stem) (**Figure 5A**). Besides, soil water holding capacity, porosity and soil aeration could also influence chemical component contents. There were positive correlations between moisture conversion coefficient, soil bulk density and most of the chemical components, and the water-holding capacity, field capacity, total porosity showed (highly) significant negative correlations with most of the chemical components. So tighter soil conditions may be associated with medicinal components accumulation, and moisture capacities may be negatively related to plant secondary accumulation for most of the medicinal components (**Figure 5B**). Moreover, P and K in rhizosphere soil showed positive correlations with plant height, ground diameter and biomass accumulation, and biomass accumulation showed (highly) significant negative correlations with most of the medicinal components, which was consistent with the “trade-off” hypothesis between biomass accumulation and secondary metabolites. TC content in stems and leaves showed positive correlations with flavonoid and phenolic acid contents, while N, P and K contents always showed negative correlations with chemical component contents in most of the organs (**Figure 5C**). In summary, looser soils were associated with better growth conditions. The negative correlations between medicinal components and growth status (nutrient contents) were consistent with the “trade-off” strategy of *S. glabra* seedlings development, which needs targeted nutrient additions and other studies in future research.

#### 3.4.2 Principal component analysis and linear-regression analysis

PCA was performed on three strategies: (1) Growth + plant/soil nutrients, (2) Growth + chemical components, and (3) All combined indices. For each strategy, two principal components (PC1 and PC2) were extracted, with cumulative contribution rates ranging from 65% to 100% (in total) (**Table 4**). In the “Growth and plant/soil nutrient contents” strategies, PC1 existed the highest loadings on TK contents in all organs and leaf TP/root TC contents, while TN/TC in rhizosphere soil and TP in root/stem contributed most, indicating that K was significant in *S. glabra*, and rhizosphere soil nutrients were also important (especially C and N); In the “Growth and chemical component contents” group, leaf phenol contents and root flavonoid/rosmarinic acid contents contributed most in PC1, while isofraxidin/rosmarinic acid and flavonoid contents in stem had the highest loading values in PC2, indicating that the PC1 and PC2 in this analysis strategy referred to the accumulation of flavonoid and isofraxidin/rosmarinic acid contents; As for all the indices, N content in plant, rhizosphere soil TK content, and moisture-related soil physical properties (especially water holding capacity) had the highest loadings, while plant aboveground growth status and some chemical components in root and stem reached the highest loadings, speculating that soil moisture also highly correlated to the growth and development of understory *S. glabra* (**Table 5**). After calculating the comprehensive PC scores, we found that the performances of *S. glabra* under CQS system in “All indices” and “Growth and plant/soil nutrient contents” strategies were all the highest among all the systems, while CIS showed the highest comprehensive scores in “Growth and chemical component contents” strategy (**Table 6**). Besides, the results of linear-regression analysis also showed that there were significant negative correlations between plant biomass and medicinal components contents in most of the organs and components (except for isofraxidin in root) (**Supplementary Table 9**). Therefore, *S. glabra* seedlings under CQS system had best growth and nutrient components performances among the three systems, and CIS system could better enhance its medicinal quality. There was a trade-off relationship between growth-medicinal quality for understory *S. glabra* seedlings.

**Table 4:** Principal component extraction of all the indices for *S. glabra* under different agroforestry systems.

Indices analysis	PC	Initial eigenvalue			Extract the sum of squared loads		
		Total	Variance percentage	Accumulation%	Total	Variance percentage	Accumulation%
All indices	1	32.74	72.76	72.76	32.74	72.76	72.76
	2	12.26	27.24	100.00	12.26	27.24	100.00
Growth and plant/soil nutrient contents	1	14.22	67.72	67.72	14.22	67.72	67.72
	2	6.78	32.28	100.00	6.78	32.28	100.00
Growth and chemical component contents	1	12.15	71.49	71.49	12.15	71.49	71.49
	2	4.85	28.51	100.00	4.85	28.51	100.00

**Table 5:** Factor loading table of all the indices for *S. glabra* under different agroforestry systems.

Indices	Loading (all indices)		Loading (growth and plant soil nutrient contents)		Loading (growth and chemical component contents)	
	PC1	PC2	PC1	PC2	PC1	PC2
Plant height	0.752	0.659	0.93	-0.368	-0.858	0.513
Ground diameter	0.598	0.801	0.832	-0.554	-0.732	0.681
Root dry weight	0.809	0.588	0.959	-0.282	-0.902	0.433
Stem dry weight	0.957	-0.292	0.803	0.595	-0.889	-0.459
Leaf dry weight	0.861	0.509	0.982	-0.191	-0.938	0.346
Moisture conversion coefficient	-0.987	0.159	–	–	–	–
Soil bulk density	-0.987	0.160	–	–	–	–
Mass moisture content	0.985	-0.171	–	–	–	–
Volumetric moisture content	0.992	-0.129	–	–	–	–
Soil water storage capacity	0.992	-0.129	–	–	–	–
Maximum water-holding capacity	0.979	-0.204	–	–	–	–
Capillary moisture capacity	0.925	-0.379	–	–	–	–
Field capacity	0.985	-0.171	–	–	–	–
Capillary porosity	0.779	-0.627	–	–	–	–
Non-capillary porosity	0.773	0.635	–	–	–	–
Total porosity	0.975	-0.220	–	–	–	–
Soil permeability	0.920	-0.392	–	–	–	–
Rhizosphere soil TC content	0.280	-0.960	-0.058	0.998	–	–
Rhizosphere soil TN content	0.346	-0.938	0.011	1.000	–	–
Rhizosphere soil TP content	0.860	0.509	0.982	-0.191	–	–
Rhizosphere soil TK content	1.000	0.009	0.945	0.327	–	–
Root TC content	0.946	0.325	1.000	0.011	–	–
Stem TC content	0.575	0.818	0.816	-0.578	–	–
Leaf TC content	0.025	1.000	0.359	-0.933	–	–
Root TN content	0.976	-0.218	0.846	0.533	–	–
Stem TN content	0.997	-0.074	0.915	0.404	–	–
Leaf TN content	0.999	0.039	0.955	0.298	–	–
Root TP content	0.439	-0.898	0.112	0.994	–	–
Stem TP content	0.589	-0.808	0.284	0.959	–	–
Leaf TP content	0.887	0.463	0.990	-0.139	–	–
Root TK content	0.892	0.451	0.992	-0.126	–	–
Stem TK content	0.986	0.168	0.985	0.172	–	–
Leaf TK content	0.973	0.230	0.994	0.109	–	–
Root total flavonoid content	-1.000	-0.001	–	–	0.984	0.179
Stem total flavonoid content	-0.700	0.714	–	–	0.560	0.828
Leaf total flavonoid content	-0.784	-0.621	–	–	0.882	-0.470
Root chlorogenic acid content	-0.982	0.191	–	–	0.931	0.364
Stem chlorogenic acid content	-0.978	0.209	–	–	0.924	0.382
Leaf chlorogenic acid content	-0.931	-0.364	–	–	0.982	-0.191
Root isofraxidin content	0.434	0.901	–	–	-0.589	0.808
Stem isofraxidin content	-0.623	0.782	–	–	0.472	0.882
Leaf isofraxidin content	-0.927	-0.374	–	–	0.980	-0.201
Root rosmarinic acid content	-0.988	0.157	–	–	0.943	0.332
Stem rosmarinic acid content	-0.633	0.775	–	–	0.483	0.876
Leaf rosmarinic acid content	-0.990	-0.142	–	–	0.999	0.038

**Table 6:** Principal component score for *S. glabra* under different agroforestry systems.

Indices analysis	Agroforestry systems	Scores			Order
		PC1	PC2	Comprehensive	
All indices	CS	3.02	-3.60	1.22	2
	CIS	-6.60	0.20	-4.75	3
	CQS	3.58	3.40	3.53	1
Growth and plant/ soil nutrient contents	CS	0.58	2.98	1.35	2
	CIS	-4.03	-1.15	-3.10	3
	CQS	3.45	-1.83	1.74	1
Growth and chemical component contents	CS	-1.17	-2.43	-1.53	3
	CIS	3.92	0.58	2.97	1
	CQS	-2.75	1.85	-1.44	2

## 4 Discussion

The “Trade-off” strategy for growth and chemical component contents in *S. glabra* reflected an important balance for understory medicinal plants (between yield and medicinal quality). Similar “growth–secondary metabolism-nutrients” relationships have been reported in various medicinal plants (like *Ophiopogon japonicus* (L. f.) Ker Gawl., *Andrographis paniculata* (Burm. f.) Wall. ex Nees, etc.), where external treatments would change the balance of their growth and metabolism status (Jian et al., 2021; Cai et al., 2025). In the following discussions, we would deeply analyze how the different upper forest systems modulate this balance for understory *S. glabra* seedlings.

### 4.1 Different mixed plantations of broad-leaf tree species significantly influence *S. glabra* growth and quality

Our research indicated that the growth status and biomass accumulation of *S. glabra* were the best in the CQS system rather than CS and CIS systems, while CIS systems suppressed *S. glabra* growth status, which did not fully conform to the hypothesis. Although proper mixed plantations of broad-leaf tree species could improve soil nutrients (carbon, nitrogen, etc.) and some key enzyme activities of (non) rhizosphere soil (Adnan and Hölscher, 2012; Ndiaye et al., 2025), the choice of mixed broadleaf species critically determined understory plants performances.

Why did CQS system promote the growth of understory *S. glabra*? Previous studies showed that it was a kind of fast-growth species with strong natural regeneration abilities, which could be used in the agroforestry systems in India or some other countries (Singh et al., 2015). The mixed plantations of *Q. griffithii* could promote the growth of other tree species (e.g., *Eucalyptus*) (Mo et al., 2022), and the soil C content of *C. lanceolata* forest could be significantly improved in the 0–20 cm and 20–40 cm soil layers after *Q. griffithii* mixed-plantations (Ming et al., 2019). Therefore, the improvement in soil organic matter would be the main reason for the growth promotion of understory plants. Other studies have shown that when *Q. griffithii* mix-planted with *C. lanceolata*, aldehyde was the main chemical component in its leaves and drop litters, and some aldehyde had the function of reducing harmful microorganisms in the soil, which may be closely related to the growth of soil properties and development of understory plants (Wen et al., 2023; Wang et al., 2024b). Thus, the mixed plantation of *Q. griffithii* could improve the growth of understory *S. glabra* seedlings.

Why did CIS suppress the growth of understory *S. glabra*? The most organs and drop litters of *I. verum* had abundant alkaloids, tannins, and other chemical components (Patra et al., 2020), which may cause complicated allelopathic effects on the understory plant. Similarly, the extractions of native species would inhibit the growth and biomass accumulation, which performed inhibitory allopathic effects and the risk of toxicity (Ghani et al., 2023). Osmotic effects, water-soluble allelopathic compounds and specific ion toxicities were the main reasons which would influence the status of understory plant growth and developments (Halvorson et al., 2017). Some specific components (like organic acids) would release from drop litters with eluviation process, and influence the plant antioxidant system (Wang et al., 2024a).

In summary, proper mixed plantations of broad-leaf species could possibly enhance the growth status of understory medicinal plants. However, the soil and litter in some mixed forests may contain chemical substances that inhibit the growth of plants in the forest, which requires more in-depth exploration.

### 4.2 Nutrient allocation of *S. glabra* could be mediated by different factors of upper forests

Our research showed that the leaves had more nutrient accumulation than the roots and stems, which was consistent with our hypothesis and was also similar to various plants. Similarly, TC content was higher in stems than in roots in December, while TN and TP content showed the opposite trend (leaf>stem) at the same time (Luo et al., 2022). Nutrient status and accumulation characteristics often show different trends than in the growth season, which may be related to nutrient transportation and storage (Ma et al., 2024). In addition, the nutrient status in the CQS systems in most of the vegetative organs showed more advantages than the other two systems, which was consistent with the hypothesis and probably related to leaf litter, root interactions, and many other aspects. Intercropping may enhance the nutrient absorption from the transformation between different plant species via spatial niche differentiation and facilitation effects about root spatial distribution and specific root exudates and interaction process (Bordron et al., 2021; Homulle et al., 2022). In our study, the improved nutrient accumulation under mixed forests may reflect such complementarity, as the deeper soil layers would provide more nutrients to upper forests, while *S. glabra* benefits from enhanced surface soil P and K availability (causing the differences of nutrient distributions among different species). Besides, the decomposition of drop litters also highly correlated with soil nutrient contents and nutrient redistribution processes. Compared with pure forests, the understory plant of broadleaf-conifer mixed forests had higher N and P content than coniferous forests (and mainly accumulated in leaf) (Pang et al., 2020); SOC (soil organic carbon) content was higher in the *C. lanceolata*-*Q. griffithii* mixed forest than *C. lanceolata* pure forest, which might be related to more leaf litter and complicated reactions of plant roots and secretions in the mixed forest (Hu et al., 2022). The decomposition trend and nutrient release pattern from the leaf litters of selected agroforestry species indicated the potential of these leaf litters to offer nutrients on a sustainable basis in an agroforestry system (Akinyele and Donald-Amaeshi, 2021). Mixed broad-leaved and coniferous forests had greater bulk soil C stocks than pure broad/needled-leaved forests, and the humus layer of the mixed broad-leaved and needle-leaved forests was often thicker, with greater litter yield, decomposition rate, and organic carbon content in the soil can be significantly increased (Zhang et al., 2023; Li et al., 2025b). After the mixture planting of *Q. griffithii* and *C. lanceolata*, the leaf litter and surface humus layer could produce more available nutrients, which could be better absorbed and utilized by the understory plants, thereby enhancing the accumulation of nutrients in the plants.

In addition, root-related effects were also correlated with the nutrient absorption of understory plants, such as an increase in fine root amounts and nutrient acquisition improvement (Xiang et al., 2015). For example, *Asparagus cochinchinensis* (Lour.) Merr. has a higher phosphorus content in underground parts under mixed forest than in pure forest (Wang et al., 2024b); Although the nutrient content of mixed forest soil can be increased compared with pure forests, the root systems of the upper forest also require a large amount of nutrients to maintain the growth and development of trees (nutrient and ecological competition) (Jose et al., 2006). Therefore, different agroforestry systems would influence the soil properties and the relationships between the forest and medicinal plants, and the nutrient absorption of the plants under the forest would be affected.

### 4.3 Trade-off between growth and chemical component contents in *S. glabra* under different agroforestry systems

Our study revealed that total flavonoid and chlorogenic acid contents of *S. glabra* were the highest under CIS system (despite its suppressed growth), while isofraxidin and rosmarinic acid were the highest under CQS system (with better growth and biomass performances). Thus, mixed forest systems (CIS and CQS) outperformed than pure CS system, which was also consistent with our hypothesis.

Similarly, the saponin content of *P. notoginseng* could be improved after intercropping with *P. orientalis* and *S. wallichii*, and some specific root exudates could inhibit the growth of harmful bacteria (like *Fusarium oxysporum*), improve the soil environment, and the yield of *P. notoginseng* (Wang et al., 2025); The total flavonoid and kaempferol contents of *Tetrastigma hemsleyanum* Diels & Gilg increased in the understory situation than after planting into containers, which would be related to the effects of interactions between the upper forest and their roots (Hu et al., 2023); and the total polyphenol contents of six kinds of medicinal plants (*Aegopodium podagraria* L., *Rubus fruticosus* L., *Rubus idaeus* L., *Galium aparine* L., *Stachys sylvatica* L., and *Urtica dioica* L.) were closely related to light density. High C/N ratio and pH in the upper layer soil were also important for phenolic compounds (Jaroszewicz et al., 2024); *Dendrobium officinale* Kimura & Migo had higher flavonoid contents when planted in the wild environment than in the greenhouse and bionic environments, and flavonoid content was closely related to soil pH and nitrogen/phosphorus content (Yuan et al., 2020). Our study also showed a similar trend of total flavonoid content in *S. glabra* seedlings, indicating that soil C and N contents would influence the chemical composition of the medicinal plants.

Moreover, the allelopathic effects of the different forests were also important. For example, studies have shown that shikimic acid (a type of phenolic acid) is the main component of *I. verum*, which might influence seed germination and plant growth, causing a stressful environment (Aniya et al., 2020). In addition, shikimic acid addition can promote the biosynthesis of jujube fruits (Jiang et al., 2025). Many studies have found that biomass (growth status) often shows negative correlations with chemical component content, and some specific compounds would accumulate under stressful environments (with lower yield and growth status). For example, root biomass is negatively correlated with the total flavonoid content of *Stellaria dichotoma* L. under different moisture content conditions (Zhang et al., 2017). In summary, the selection of the upper forest layer also had significant impacts on the targeted cultivation of understory medicinal plants, which was closely related to the growth status and medicinal quality. Although canopy densities among the three agroforestry systems were not significantly different, the direct measurements of understory photosynthetically active radiation (PAR) and some other characteristics of lights (such as light intensity, light quality, sunfleck dynamics, etc.) were not conducted. Therefore, the interactions of light conditions and soil-driven effects between different understory spaces should be laid more emphasis in the future research.

### 4.4 Mixed plantation could better improve soil physio-chemical properties and rhizosphere C and P availability of *S. glabra*

Our research found that rhizosphere soil had higher TC and TP contents than non-rhizosphere soil in all three systems, and for non-rhizosphere soil, TC, TN, and TP contents were higher in the 0–20 cm soil layer than in the 20–40 cm layer soil, which was related to “nutrient surface accumulation” effects. Drop litters and natural sedimentation process would bring organic matter and nitrogen to the surface soil, which could be found in several needled-leaved and broad-leaved forests such as *Fokienia hodginsii* (Dunn) A. Henry & H. H. Thomas*-P. massoniana* mixed forest, *Castanopsis hystrix* Hook. f. & Thomson ex A. DC. forest, etc (Wang et al., 2021; Liu et al., 2022). Proper intercropping (like *Terminalia chebula* Retz. based agroforestry system) could enhance soil N content rather than the mono-cropping system, and most forms of nitrogen (including NH_4_^+^-N, NO_3_^--^N, and total inorganic nitrogen) contents were higher in the upper layer soil (0~15 cm) than in the deeper layer soil (15~30 cm) (Kumar et al., 2020). These findings are similar to those of our study. For TP and TK contents, TP content was higher in the 0–20 cm soil layer than in the 20–40 cm soil layer, but TP content in CQS system and TK contents in all the three systems showed the opposite trends. The activities of acid phosphatase and other soil enzymes would increase after mixed with broad-leaf tree species, which could transform P from unavailable forms into phosphate ions (which could be absorbed by microorganisms and root systems better) (Huang et al., 2022). Meanwhile, some beneficial microbial (like saprotrophic fungi, arbuscular mycorrhizal fungi and ectomycorrhizal) would increase in rhizosphere soil and the availability of soil nutrients could be increased (Li et al., 2021). So the P contents in broad-leaf mixed *C. lanceolata* forest were higher than the pure forest. Besides, in the agroforestry systems of *Ginkgo biloba* L. and other crops, P and K contents increased with the increasing soil layer depth (Guo et al., 2020); In *Annona senegalensis* Pers. agroforestry systems, soil P and K contents increased with the increasing of soil layers, which might depend on the characteristics of nutrient uptakes (Diallo et al., 2019). Some research showed that shallow roots would absorb most of the K in the upper soil layer, and some of the K nutrient would leach to deeper soil layers, causing higher contents in deeper soil layer than the upper soil layers (Pivetta et al., 2011; Rosolem and Steiner, 2017). So K would better accumulate in the deeper soil layers. In conclusion, nutrient content and distribution characteristics in rhizosphere and non-rhizosphere soil were related to tree species, cultivation methods, and many other factors in different agroforestry systems, and the nutrient content of rhizosphere soil often determines the absorption and accumulation of nutrients by understory plants.

### 4.5 Trade-off between yield and medicinal quality: roles of soil compaction and nutrient availability of *S. glabra*

The growth status and chemical quality of medicinal plants are strongly related to nutrients and soil properties. In this study, moisture holding capacity and soil looseness were closely related to the growth of seedlings and the biosynthesis of medicinal components, and medicinal component content might decrease in accordance with the accumulation of nutrient elements, which was similar to the hypothesis in this study and many previous studies. For example, in a rubber agroforestry system, soil moisture content was positively correlated with C and N content, and soil nutrients decreased with an increase in plant species and nutrient competition (Wu et al., 2020). The total flavonoid content of mango and total phenol content of guava and avocado were moderately negatively correlated with the soil moisture content and regional rainfall (Omwango et al., 2024). Some studies have also shown that mild improper planting methods or stressful environments would promote flavonoid biosynthesis in medicinal plants. Similarly, the icariin-flavonoid content of *Epimedium pubescens* Maxim. showed negative correlations with potassium and positive correlations with nitrogen in some specific growth stages, and the available potassium content in the soil also showed similar correlations with plant flavonoid content (An et al., 2025). Certain nutrient stress environments can also improve the quality of medicinal plants. Low-nitrogen treatment promotes the growth and development of flowers and increases flavonoid biosynthesis in snow *chrysanthemum* (Li et al., 2023b). In summary, proper cultivation methods and nutrient addition plans are necessary for understory cultivation of *S. glabra* seedlings.

## 5 Conclusion

We showed that the growth, biomass, and the contents of isofraxidin and rosmarinic acid performed best in CQS systems (*C. lanceolata*-*Q. griffithii+S. glabra*) of the three understory systems. Among all the systems, leaf was the dominant organ for nutrient accumulation, while root accumulated the most chemical components. Although CIS system (*C. lanceolata-I. verum+S. glabra*) suppressed the growth and biomass accumulation of *S. glabra* seedlings, the total flavonoid and chlorogenic acid also accumulated best. CQS improved soil carbon and phosphorus availability, while CIS increased water-holding capacity but also soil compaction. Many nutrient contents showed negative correlations with plant chemical component contents. Thus, a clear trade-off exists between yield and medicinal quality. CQS is recommended for maximizing yield and specific phenolic acids, while CIS is suitable for total flavonoid accumulation (with proper cultivation methods). Future research should investigate the allelopathic mechanisms underlying these system-specific responses and develop targeted cultivation practices to balance growth and quality in understory *S. glabra* production.

## Data Availability

The raw data supporting the conclusions of this article will be made available by the authors, without undue reservation.
