# Supplementary material for: Trade-off between growth, nutrient absorption and medicinal quality of Sarcandra glabra (Thunb.) Nakai under different Chinese fir-based agroforestry systems
Source: Front Plant Sci. 2026 Jul 1;17:1868306. doi: 10.3389/fpls.2026.1868306 (PMC13368759; doi:10.3389/fpls.2026.1868306)
Supplement: Supplementary file 1 [file Table1.docx]

**Supplementary Tables**

**Supplementary Table S1 *F*-value and *P*-value of aboveground growth indexes of *S. glabra* under different planting patterns**

| **Index** | **Height** | **Ground diameter** |
| --- | --- | --- |
| *F*-value | 17.184 | 15.536 |
| *P*-value | <0.01 | <0.01 |

**Supplementary Table S2 *F*-value and *P*-value of biomass accumulation in root, stem and leaf of *S. glabra* under different planting patterns**

| **Fresh weight** | ***F*-value** | ***P*-value** | **Dry weight** | ***F*-value** | ***P*-value** | **Moisture content** | ***F*-value** | ***P*-value** |
| --- | --- | --- | --- | --- | --- | --- | --- | --- |
| Root | 20.194 | 0.002 | Root | 11.500 | 0.009 | Root | 105.548 | <0.01 |
| Stem | 36.069 | <0.01 | Stem | 126.201 | <0.01 | Stem | 4.462 | 0.065 |
| Leaf | 13.363 | 0.006 | Leaf | 5.146 | 0.05 | Leaf | 65.330 | <0.01 |

| **Supplementary Table S3 *F*-value and *P*-value of nutrient content in root, stem and leaf of *S. glabra* under different planting patterns** | | | | | |
| --- | --- | --- | --- | --- | --- |
| **TC content** | ***F*-value** | ***P*-value** | **TN content** | ***F*-value** | ***P*-value** |
| Root | 6.361 | <0.05 | Root | 44.087 | <0.01 |
| Stem | 0.876 | 0.464 | Stem | 42.735 | <0.01 |
| Leaf | 2.190 | 0.193 | Leaf | 27.266 | <0.01 |
| **TP content** | ***F*-value** | ***P*-value** | **TK content** | ***F*-value** | ***P*-value** |
| Root | 57.802 | <0.01 | Root | 259.098 | <0.01 |
| Stem | 34.875 | <0.01 | Stem | 5.627 | <0.05 |
| Leaf | 1.998 | 0.216 | Leaf | 50.602 | <0.01 |

**Supplementary Table S4 *F*-value and *P*-value of nutrient** **stoichiometric ratio in root, stem and leaf of *S. glabra* under different planting patterns**

| **TC/TN** | ***F*-value** | ***P*-value** | **TC/TP** | ***F*-value** | ***P*-value** | **TN/TP** | ***F*-value** | ***P*-value** |
| --- | --- | --- | --- | --- | --- | --- | --- | --- |
| Root | 24.069 | <0.01 | Root | 8.948 | <0.05 | Root | 29.674 | <0.01 |
| Stem | 42.397 | <0.01 | Stem | 8.398 | <0.05 | Stem | 47.590 | <0.01 |
| Leaf | 116.763 | <0.01 | Leaf | 1.774 | 0.248 | Leaf | 2.074 | 0.207 |
| **TN/TK** | ***F*-value** | ***P*-value** | **TK/TP** | ***F*-value** | ***P*-value** |  |  |  |
| Root | 21.578 | <0.01 | Root | 220.905 | <0.01 |  |  |  |
| Stem | 16.831 | <0.01 | Stem | 10.797 | <0.05 |  |  |  |
| Leaf | 2.720 | 0.144 | Leaf | 6.745 | <0.05 |  |  |  |

| **Supplementary Table S5 *F*-value and *P*-value of chemical component contents in root, stem and leaf of *S. glabra* under different planting patterns** | | | | | |
| --- | --- | --- | --- | --- | --- |
| **Total flavonoid content** | ***F*-value** | ***P*-value** | **Chlorogenic acid content** | ***F*-value** | ***P*-value** |
| Root | 8141.182 | <0.01 | Root | 163.263 | <0.01 |
| Stem | 1.765 | 0.250 | Stem | 320.424 | <0.01 |
| Leaf | 3.493 | 0.099 | Leaf | 45.091 | <0.01 |
| **Isofraxidin content** | ***F*-value** | ***P*-value** | **Rosmarinic acid content** | ***F*-value** | ***P*-value** |
| Root | 98.817 | <0.01 | Root | 278.859 | <0.01 |
| Stem | 4540.900 | <0.01 | Stem | 481.739 | <0.01 |
| Leaf | 90.693 | <0.01 | Leaf | 1020.005 | <0.01 |

| **Supplementary Table S6 *F*-value and *P*-value of soil physical characteristics in different layers of soil under different *S. glabra* understory planting patterns** | | | | | | | | | | | | | | | | | |
| --- | --- | --- | --- | --- | --- | --- | --- | --- | --- | --- | --- | --- | --- | --- | --- | --- | --- |
| Moisture content | | | | |  | Soil bulk density | | | | |  | Mass moisture content | | | | | |
| 0–20 cm | |  | 20–40 cm | |  | 0–20 cm | |  | 20–40 cm | |  | 0–20 cm | |  | 20–40 cm | |  |
| *F-*value | *P-*value |  | *F-*value | *P-*value |  | *F-*value | *P-*value |  | *F-*value | *P-*value |  | *F-*value | *P-*value |  | *F-*value | *P-*value |  |
| 16.111 | 0.004 |  | 6.683 | 0.030 |  | 7.896 | 0.021 |  | 19.001 | 0.003 |  | 15.817 | 0.004 |  | 6.177 | 0.035 |  |
| Volumetric moisture content | | | | |  | Soil water storage capacity | | | | |  | Maximum water-holding capacity | | | | | |
| 0–20 cm | |  | 20–40 cm | |  | 0–20 cm | |  | 20–40 cm | |  | 0–20 cm | |  | 20–40 cm | |  |
| *F-*value | *P-*value |  | *F-*value | *P-*value |  | *F-*value | *P-*value |  | *F-*value | *P-*value |  | *F-*value | *P-*value |  | *F-*value | *P-*value |  |
| 34.239 | 0.001 |  | 2.191 | 0.193 |  | 34.274 | 0.001 |  | 2.198 | 0.192 |  | 5.117 | 0.050 |  | 57.311 | 0.000 |  |
| Capillary moisture capacity | | | | |  | Field capacity | | | | |  | Capillary porosity | | | | | |
| 0–20 cm | |  | 20–40 cm | |  | 0–20 cm | |  | 20–40 cm | |  | 0–20 cm | |  | 20–40 cm | |  |
| *F-*value | *P-*value |  | *F-*value | *P-*value |  | *F-*value | *P-*value |  | *F-*value | *P-*value |  | *F-*value | *P-*value |  | *F-*value | *P-*value |  |
| 11.046 | 0.010 |  | 21.394 | 0.002 |  | 15.817 | 0.004 |  | 6.177 | 0.035 |  | 18.302 | 0.003 |  | 5.456 | 0.045 |  |
| Noncapillary porosity | | | | |  | Total porosity | | | | |  | Soil permeability | | | | | |
| 0–20 cm | |  | 20–40 cm | |  | 0–20 cm | |  | 20–40 cm | |  | 0–20 cm | |  | 20–40 cm | |  |
| *F-*value | *P-*value |  | *F-*value | *P-*value |  | *F-*value | *P-*value |  | *F-*value | *P-*value |  | *F-*value | *P-*value |  | *F-*value | *P-*value |  |
| 4.096 | 0.076 |  | 2.841 | 0.135 |  | 3.509 | 0.098 |  | 42.533 | 0.000 |  | 10.266 | 0.012 |  | 0.477 | 0.642 |  |

| **Supplementary Table S7 *F*-value and *P*-value of nutrient contents in different layers of soil under different *S. glabra* understory planting patterns** | | | | | | | | | | |
| --- | --- | --- | --- | --- | --- | --- | --- | --- | --- | --- |
| **Indexes** | **0**–**20 cm** | |  | **Indexes** | **20**–**40 cm** | |  | **Indexes** | **Rhizosphere** | |
|  | ***F-*value** | ***P-*value** |  |  | ***F-*value** | ***P-*value** |  |  | ***F-*value** | ***P-*value** |
| TC content | 2.716 | 0.145 |  | TC content | 8.587 | <0.05 |  | TC content | 21.009 | <0.01 |
| TN content | 8.080 | <0.05 |  | TN content | 115.170 | <0.01 |  | TN content | 18.440 | <0.01 |
| TP content | 77.357 | <0.01 |  | TP content | 51.947 | <0.01 |  | TP content | 8.143 | <0.05 |
| TK content | 32.303 | <0.01 |  | TK content | 6.327 | <0.05 |  | TK content | 42.742 | <0.01 |

| **Supplementary Table S8 *F*-value and *P*-value of nutrient stoichiometric ratio in different layers of soil under different *S. glabra* understory planting patterns** | | | | | | | | | | |
| --- | --- | --- | --- | --- | --- | --- | --- | --- | --- | --- |
| **Indexes** | **0**–**20 cm** | |  | **Indexes** | **20**–**40 cm** | |  | **Indexes** | **Rhizosphere** | |
|  | ***F-*value** | ***P-*value** |  |  | ***F-*value** | ***P-*value** |  |  | ***F-*value** | ***P-*value** |
| TC/TN | 0.003 | 0.997 |  | TC/TN | 19.578 | <0.01 |  | TC/TN | 1.072 | 0.400 |
| TC/TP | 19.641 | <0.01 |  | TC/TP | 19.457 | <0.01 |  | TC/TP | 7.613 | <0.05 |
| TN/TP | 43.099 | <0.01 |  | TN/TP | 27.829 | <0.01 |  | TN/TP | 6.085 | <0.05 |
| TN/TK | 3.096 | 0.119 |  | TN/TK | 6.226 | <0.05 |  | TN/TK | 18.257 | <0.01 |
| TK/TP | 30.645 | <0.01 |  | TK/TP | 30.576 | <0.01 |  | TK/TP | 1.416 | 0.313 |

**Supplementary Table S9 Linear regression analysis between biomass accumulation and chemical component contents in different organs of *S. glabra***

| **Biomass** | **Chemical component content** | **Correlation coefficient** | **R^2^** | **Sig.** |
| --- | --- | --- | --- | --- |
| Leaf biomass | leaf total flavonoid content | -0.573 | 0.328 | 0.053 |
| Leaf biomass | leaf chlorogenic acid content | -0.732 | 0.470 | 0.012 |
| Leaf biomass | leaf isofraxidin content | -0.770 | 0.534 | 0.008 |
| Leaf biomass | leaf rosmarinic acid content | -0.732 | 0.470 | 0.012 |
| Stem biomass | Stem total flavonoid content | -0.505 | 0.255 | 0.083 |
| Stem biomass | Stem chlorogenic acid content | -0.992 | 0.981 | 0.000 |
| Stem biomass | Stem isofraxidin content | -0.814 | 0.615 | 0.004 |
| Stem biomass | Stem rosmarinic acid content | -0.816 | 0.619 | 0.004 |
| Root biomass | Root total flavonoid content | -0.722 | 0.453 | 0.014 |
| Root biomass | Root chlorogenic acid content | -0.608 | 0.279 | 0.041 |
| Root biomass | Root isofraxidin content | 0.730 | 0.467 | 0.013 |
| Root biomass | Root rosmarinic acid content | -0.640 | 0.325 | 0.032 |
